# Role of Carbonic Anhydrase in Cerebral Ischemia and Carbonic Anhydrase Inhibitors as Putative Protective Agents

**DOI:** 10.3390/ijms22095029

**Published:** 2021-05-10

**Authors:** Irene Bulli, Ilaria Dettori, Elisabetta Coppi, Federica Cherchi, Martina Venturini, Lorenzo Di Cesare Mannelli, Carla Ghelardini, Alessio Nocentini, Claudiu T. Supuran, Anna Maria Pugliese, Felicita Pedata

**Affiliations:** 1 Department of Neuroscience, Psycology, Drug Research and Child Health (NEUROFARBA), Section of Pharmacology and Toxicology, University of Florence, 50139 Florence, Italy; irene.bulli@unifi.it (I.B.); ilaria.dettori@unifi.it (I.D.); elisabetta.coppi@unifi.it (E.C.); federica.cherchi@unifi.it (F.C.); martina.venturini@unifi.it (M.V.); lorenzo.mannelli@unifi.it (L.D.C.M.); carla.ghelardini@unifi.it (C.G.); annamaria.pugliese@unifi.it (A.M.P.); 2 Department of Neuroscience, Psycology, Drug Research and Child Health (NEUROFARBA), Section of Pharmaceutical Sciences, University of Florence, 50019 Florence, Italy; alessio.nocentini@unifi.it

**Keywords:** carbonic anhydrase, inhibitors, sulfonamide, cerebral ischemia, middle cerebral artery occlusion, ischemic acidosis

## Abstract

Ischemic stroke is a leading cause of death and disability worldwide. The only pharmacological treatment available to date for cerebral ischemia is tissue plasminogen activator (t-PA) and the search for successful therapeutic strategies still remains a major challenge. The loss of cerebral blood flow leads to reduced oxygen and glucose supply and a subsequent switch to the glycolytic pathway, which leads to tissue acidification. Carbonic anhydrase (CA, EC 4.2.1.1) is the enzyme responsible for converting carbon dioxide into a protons and bicarbonate, thus contributing to pH regulation and metabolism, with many CA isoforms present in the brain. Recently, numerous studies have shed light on several classes of carbonic anhydrase inhibitor (CAI) as possible new pharmacological agents for the management of brain ischemia. In the present review we summarized pharmacological, preclinical and clinical findings regarding the role of CAIs in strokes and we discuss their potential protective mechanisms.

## 1. Introduction

Ischemic stroke is the second most common cause of death and a major cause of long-term disability worldwide and it is thus considered a global burden. It is characterized by early glutamate-mediated excitotoxicity, followed by a chronic secondary damage caused by the activation of resident immune cells, i.e., microglia, and the production of inflammatory mediators [1]. Unfortunately, despite advances in understanding of the pathophysiology of cerebral ischemia and the development of more than 1000 molecules with brain-protective effects in animal models, drugs so far have failed to be efficacious during clinical trials [2]. The only successful pharmacological strategy approved to date consists in the intravascular administration of tissue plasminogen activator (t-PA), a thrombolytic treatment to dissolve the intravascular clot. However, t-PA must be administered within the first 4–4.5 h after stroke onset and can result in increased risk of hemorrhagic transformation [3]. Because of its narrow therapeutic time-window and its important side effects, thrombolytic application is very limited in clinical practice [4]. Therefore, the search for successful therapeutic strategies for acute ischemic stroke still remains one of the major challenges in clinical medicine. Ischemic stroke accounts for 80% of all stroke cases [5] and is caused by the occlusion of a major cerebral artery by a thrombus or an embolism. The occlusion leads to a reduction of cerebral blood flow rate, a condition of hypoxia and glucose deprivation (oxygen, glucose deprivation: OGD) and subsequent tissue damage in the affected region [6].

In this hypoxic/ischemic condition, the oxidative phosphorylation of glucose is impaired, thus most energy derives from the anaerobic glycolytic pathway which leads to protons and lactate accumulation and consequent ambient acidification [7,8]. Indeed, during cerebral ischemia, brain pH falls from ~7.2 to below 6.5 within minutes after stroke onset [9,10]. In hypoxic/anoxic conditions, in vitro studies have shown a decrease in pH in neurons and glial cells [11]. Brain acidosis itself causes neuronal injury by generating free radicals, affecting glutamate reuptake, glial cell activation and neuronal apoptosis [12,13] and exacerbates ischemic brain injury [14,15] leading to cerebral infarction such as edema and blood-brain barrier (BBB) dysfunction [16,17].

Since the role of carbonic anhydrases (CAs) is to catalyze the reversible hydratation of carbon dioxide into a bicarbonate ion and a proton (CO_2_ + H_2_O ⇆ HCO_3_^−^ + H^+^), thus playing a pivotal role in pH regulation and metabolism [18,19], this review will highlight the role of carbonic anhydrase as a possible therapeutic target in brain ischemia. In particular, the role of carbonic anhydrase inhibitors (CAIs) for the maintenance of pH homeostasis following an ischemic insult will be discussed.

## 2. Carbonic Anhydrase Inhibitors (CAIs) as Possible Therapeutics in the Central Nervous System Pathologies

**Figure 1:**
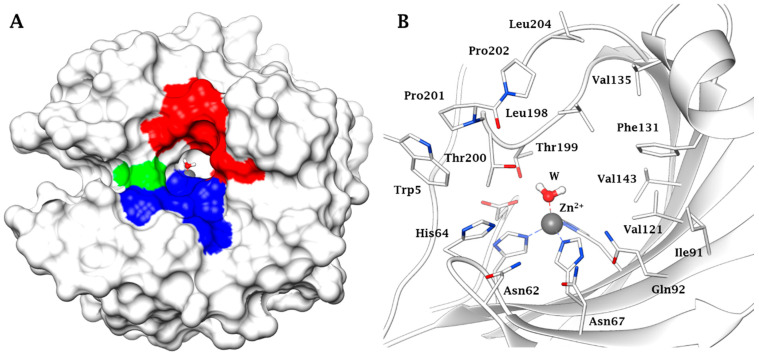
(**A**) Surface representation of human (h) isoform carbonic anhydrase (hCA II) (pdb 3KKX). The hydrophobic half of the active site is colored in red (Ile91, Val121, Phe131, Val135, Val143, Leu198, Pro201, Pro202, Leu204), the hydrophilic one in blue (Asn62, Asn67, Glu69, Gln92, His94). His64, the proton shuttle residue, is in green. (**B**) Active site view of hCA II. The zinc ion, represented as grey sphere, is tetrahedrally coordinated to residues His94, His96 and His119 and to a water molecule/hydroxide ion as fourth ligand.

CAs are a family of ubiquitous metalloenzymes present in most organisms all over the phylogenetic tree [19]. To date, eight CA classes are known: α-, β-, γ-, δ-, ζ-, η-, θ-, and ι-CAs [20], the last three recently discovered [21,22,23]. CAs present in animals belong to α-class, and a large number of α-CA isoforms has been described: 15 in humans and other primates, and 16 in other mammals, with different catalytic activity and subcellular localization [19]. The three-dimensional (3D) fold of the main CA mammalian isoform (in this specific case the human (h) isoform hCA II) is shown in Figure 1, with the hydrophobic, hydrophilic and proton transfer regions highlighted (Figure 1A), whereas the zinc coordination and the amino acid residues crucial for catalysis and inhibition are shown in detail in Figure 1B [18,19,20]. Indeed, the active site architecture of α-CAs is unique, with half of the cavity being lined with hydrophobic and the opposite half with hydrophilic amino acid residues, as observed from Figure 1. The metal ion is placed at the bottom of this cavity, and the water molecule coordinated to it plays a crucial role in the catalytic process, being activated by the zinc ion for the nucleophilic attack on the various substrates on which the CAs act, but the physiological one seems to be only CO_2_, which is hydrated to bicarbonate and protons [18,19,20].

**Figure 2:**
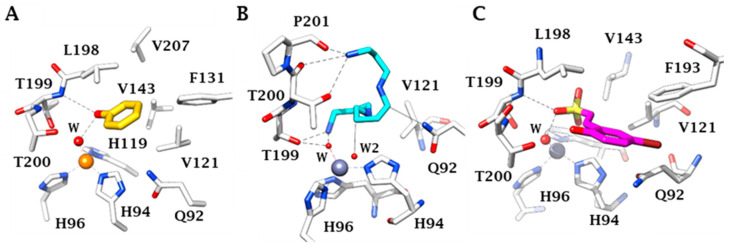
X-ray crystal structure for the adducts of hCA II with phenol (**A**), spermine (**B**) and hydrolysed sulfocoumarin (**C**), new CA inhibitory chemotyes which bind by anchoring to the zinc-coordinated water molecule. The metal ion is shown as a gold or gray sphere with its three histidine ligands and the coordinated water molecule. Amino acid residues involved in the binding of the inhibitors are also highlighted. Phenol is shown in yellow, spermine in blue and the hydrolysed sulfocoumarin in magenta.

This particular, rather large, type of active site probably is also responsible for the fact that these enzymes are inhibited by many classes of very diverse inhibitor [18]. The classical ones are the primary sulfonamides and their isosteres, such as the sulfamides and the sulfamates. They coordinate to the zinc ion as anions, in deprotonated form and some of them show low nanomolar affinity for the various CA isoforms present in vertebrates, including humans [18,19,20]. In the last decade, a variety of new chemotypes with CA inhibitory activity and with new inhibition mechanisms were discovered, some of which are independent of the metal ion found within the enzyme active site [20,21,22]. They include the anchoring to the zinc-coordinated water (for phenols, polyphenols, polyamines, sulfocoumarins, thioxocoumarins) [20,21,22]; the occlusion of the active site entrance, for coumarins and their derivatives [20,21,22]; and even compounds which bind outside the active site cavity, such as some benzoic acid derivatives [22]. The inhibition mechanisms with some of these compounds are shown in Figure 2, as determined by X-ray crystallography [20,21,22].

The mammalian central nervous system (CNS) has the highest number of CA isoforms (at least 9) among all investigated organs [19]. One of the most abundant ones is hCA II, but isoforms I, VB, VII, VIII, X, XI, XII and XIV are also present [19]. Given the wide range of CA isoform expression in the brain, CAIs have been exploited for therapeutic application in several pathological conditions of the CNS [24]. Inhibition by CAIs proved clinically useful in epilepsy [25,26,27] and in idiopathic intracranial hypertension (IIH), where the acetazolamide (ACTZ, Compound **1**, Figure 2) is one of the drugs currently used clinically [19,28]. Other possible pharmacological applications of CAIs targeting CNS isoforms include neuropathic pain [29,30], diabetes-induced BBB disruption [31,32], migraine [33], and amyloid β-induced mitochondrial dysfunction typical of Alzheimer’s disease [34,35,36].

A relationship between brain hypoxia and CA has been highlighted. It has been reported that CA II-deficient mice are more resistant to hypoxia-induced neuronal damage [37], and that blocking CA leads to a reduced neuronal apoptosis via pH stabilization [38]. Moreover, hypoxic conditions elicit the overexpression of two CA isoforms (IX and XII), through the hypoxia inducible factor [39,40]. All these findings led to the hypothesis of a possible CA relevance in brain ischemia, with CA inhibition contributing to pH homeostasis [19,41].

## 3. Role of CAIs in Brain Ischemia Preclinical Models

The first paper assessing the effect of CAIs in cerebral ischemia demonstrated that cats undergoing middle cerebral artery occlusion (MCAo) and treated with ACTZ (in five doses for a total of 500 mg intramuscularly in two days after ischemia), 8 days after ischemia induction, generated a more severe neurological deficit, larger areas of infarction and more brain swelling with respect to untreated cats. In a model of collagenase-induced striatal hemorrhage in rats, ACTZ, 50 mg/kg intraperitoneally (i.p.) administered, starting 3 h after inducing intracerebral hemorrhage, despite reducing the spike of increased intracranial pressure by presumably reducing cerebrospinal fluid production, did not improve behavioral function or did not affect lesion size up to 28 days thereafter [42].

In contrast, several investigations support a potential therapeutic role of low doses of new CAIs in strokes [43].

There are many classes of CAI, but the most investigated ones are the sulfonamides and the coumarins [18]. Examples of some of these derivatives (Compounds **1–7**), which have also been investigated for their effects in various pathologies, including brain ischemia, are shown in Figure 2.

Di Cesare Mannelli et al. (2016) evaluated the effect of several newly synthetized sulphonamide and coumarin CAIs (Compounds **2–5**, Figure 2) in the permanent MCAo (pMCAo) model of cerebral ischemia in the rat. They found that repeated subcutaneous injections (5 and 20 min after surgery) of CAIs at the dose of 1 mg/kg were able to significantly reduce the neurological deficit 24 h after pMCAo, whereas the prototypical CAI, ACTZ, 30 mg/kg subcutaneously was ineffective in reducing the neurological deficit. In addition, it has been reported that ACTZ at the dose of 100 mg/kg injected into the femoral vein 30 min after transient (1.5 h) MCAo, 22 h thereafter, reduced the infarct volume in male Wistar rats [44].

**Figure 3:**
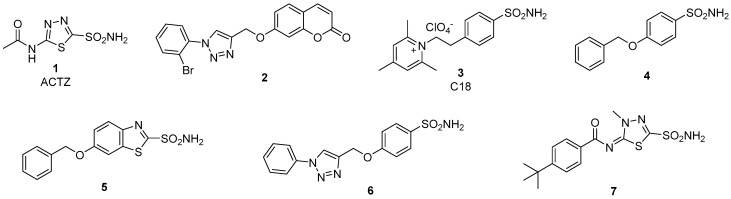
Chemical structure of CAIs **1**–**7** investigated as protective agents in cerebral ischemia. Except acetazolamide **1**, which is a CA pan-inhibitor [18], compounds **2**–**7** show a selective inhibition of some CA isoforms present in the brain, such as CA VII, or of the two isoforms overexpressed in hypoxia, CA IX and XII, for which they act as low nanomolar inhibitors.

Recently, Dettori et al. (2021) demonstrated that ACTZ and a lipophilic CA inhibitor of new generation (Compound **7**, Figure 3) [45,46,47] administered i.p. at the dose of 4.4 mg/kg and 1.0 mg/kg respectively, 5 min, 6 and 20 h after starting pMCAo in the rat, 24 h thereafter, significantly reduced the neurological deficit and the infarct volume within the cortex and striatum. At the same time after MCAo, CAIs re-established the cytoarchitecture of the ischemic cortex and striatum, counteracted neuronal loss, reduced microglia activation and partially counteracted the loss of astrocytes in the cortical and striatal ischemic areas. In the in vitro model of ischemia in hippocampal slices exposed to a severe (30 min) OGD, the same CAIs significantly delayed the appearance of anoxic depolarization (AD) induced by OGD [45]. AD is a robust neuronal depolarization demonstrated both in vivo [48] and in vitro [49,50]. AD gives rise to recurrent peri-infarct depolarization that arises at the border of the ischemic core during the first 3–4 h post-stroke [51,52,53,54,55]. AD spreading to the ischemic penumbra represents an early and critical event after ischemia that contributes to lactate accumulation [56] and reduction of tissue pH [57], thereby prolonging tissue acidosis and increasing the risk of neuronal injury [58]. AD is considered a clear sign of excitotoxic damage [48] because the sustained activation of N-methyl-d-aspartate (NMDA)-type glutamate receptors is essential to AD initiation and propagation in the ischemic penumbra. Since the ischemic penumbra is considered the most salvageable area soon after ischemia, it is well accepted that a pharmacological treatment that postpones the onset of AD helps to protect brain tissue after ischemia.

## 4. Role of CAIs in Stroke Clinical Models

Up until now, CAIs are not used in the management of cerebral ischemia. The only available clinical study exploring protection by CA inhibitors has been made in hemorrhagic stroke patients. ACTZ treatment (750 mg/day administered every 8 h), proved protective 72 h and 3 weeks after intracerebral haemorrhage improving neurological functionalities and decreasing the mortality rate in treated patients [59].

## 5. Protective Mechanisms of CAIs in Cerebral Ischemia

One of the most likely mechanisms by which CAIs can be protective in brain ischemia is the reduction of hydrogen ions and thus the maintenance of pH homeostasis. Under physiological conditions, extracellular and intracellular pH are generally maintained at ~7.3 and ~7.0, respectively [9]. Glial cells, in particular astrocytes, express high levels of CA [60,61] in order to convert neuron-derived CO_2_ into bicarbonate and protons, which are then extruded from the glial cell by a Na^+^/HCO_3_^−^ cotransporter and by monocarboxylate transporters [62]. Astrocytes have, therefore, a key role in pH regulation in the brain [63]. Extracellularly, CA is pivotal in buffering extracellular pH by recycling CO_2_ in bicarbonate and protons [61]. Cerebral ischemia causes tissue acidosis, and it is known that low pH augments the vulnerability of glia to injury induced by OGD [64]. Intracellular lactate-induced acidification of astrocytes is reduced in the presence of a non-specific CAI [60]. Moreover, neurons are particularly sensitive to pH decrease [11]. Indeed, changes in the intracellular pH may affect neurotransmitter release. Lowering pH results in increased release of dopamine [65,66], noradrenaline and serotonin from rat brain synaptosomes [66]. Glial acidosis has been shown to trigger also glial glutamate release [67] and it is well established that sustained activation of NMDA-type glutamate receptors is essential in leading to early excitotoxic neuronal death in stroke [68]. Results from Dettori et al. (2021) demonstrating that, in hippocampal OGD slices, CAIs significantly delayed the phenomenon of AD, which is strictly dependent on NMDA receptor activation, strongly supports the assertion that CAIs, by reestablishing H+ concentration during ischemia and reducing the ensuing excitatory amino acid efflux, protect from glutamate-induced early excitotoxic damage.

Moreover, since multiple CA isoforms are expressed in cerebral arteries, CAIs, by decreasing intracellular acidosis, may protect the ischemia-induced BBB breakdown in the cerebrovascular wall during MCAo. Indeed, it has been found that ACTZ (100 µM) reduces the rate of intracellular acidification in the cerebrovascular wall of isolated rat middle cerebral arteries [69] and that CAIs decrease hypoxic-mediated brain vascular leakage in a rat model of high-altitude sickness [70,71].

Although protection in vivo by CAIs against brain hypoxic/ischemic damage may be due to reduction of tissue acidosis and early glutamate excitotoxicity, protection by CAIs may also be related to different effects.

Gao et al. (2007) have demonstrated that subdural infusion of CA in rats increases cerebral vascular permeability, suggesting that it might have relevance in brain edema. In agreement, intracaudate injection of CA increases brain water content and neuronal death, whereas intracerebral injection of ACTZ (5 μL, 1 mM) reduces brain edema, neuronal death and neurological deficit 24 h after intracerebral hemorrhage in Sprague–Dawley rats [72].

ACTZ is also known to reduce the permeability of the predominant water channel in the brain, aquaporin-4, known to be involved in cerebral edema [73,74,75]. The reduction of brain edema after ischemia could also be due to the diuretic action of CAIs [24]. Actually, CAIs are currently clinically used to reduce body fluid volume in pathologies like glaucoma [76], idiopathic intracranial hypertension [28], congestive heart failure induced- or drug-induced edema [77], and to prevent high-altitude cerebral edema [78]. CAIs, by regulating the volume of body fluids and thus brain water content, may therefore alleviate cerebral edema, which contributes to poor outcomes in ischemic strokes leading to high intracranial pressure and to compression of the nervous tissue [79,80].

CAIs may be protective in ischemic stroke also by regulating the vascular tone, leading to vasodilatation of cerebral arterioles and thus to increased cerebral blood flow and oxygen supply [81,82]. Indeed, CAIs have been related to increased production of nitric oxide (NO), a vasodilator molecule [83]. Even if, in cerebral circulation, the vasodilating effect of ACTZ appears independent of NO [84], its vasodilator effect might be mediated by vascular calcium-activated potassium (KCa) channel activation [85]. Moreover, ACTZ inhibits vasoconstriction during intracellular acidification, as occurs during cerebral ischemia [69].

Finally, reduction of ischemic brain injury by CAIs may also be related to other mechanisms, since ACTZ has been reported to reduce inflammation and the production of pro-inflammatory cytokines [86,87]. Indeed, in rats exposed to high altitude and thus to hypoxic injury, ACTZ decreases mRNA expression of IL-1β, TNF-α and IFN-γ in the lung [87]. During epileptogenesis, ACTZ reduces the IL-1β, IL-6, and TNF-α mRNA levels in rat hippocampus, and diminishes proinflammatory cytokines in rat serum [86].

## 6. Conclusions

Many CA isoforms are present in the brain, where they play various functions connected with a variety of physiological and pathological processes. This is certainly due to the fact that the pH homeostasis, the signalling role of bicarbonate and the metabolic roles of these enzymes modulate a variety of such processes. Thus, considering the fact that some isoforms are overexpressed as a consequence of hypoxia, the idea of using their inhibition as a new approach for the management of cerebral ischemia has led to careful investigations over the last few years. These recent results indicate that CAIs could represent an innovative pharmacological tool for the treatment of cerebral ischemia, and may complement t-PA-based therapy in its therapeutic time-window. Although ACTZ is able to reach the nervous tissue [88], more lipophilic CAIs such as those discussed in the present review could be particularly relevant for clinical translatability, because crossing the BBB soon after ischemia may induce early neuroprotection [45]. Drug design studies of isoform-selective CAIs able to easily cross the BBB are recommended in order to develop more effective pharmacological agents. However, the main limitation at present is poor understanding of the differential role/s of various brain CA isoforms in this pathology, as there are at least 9 CAs present in the brain. Further and more detailed pharmacological studies are needed to assess if CAIs can be protective at a later time after ischemia induction.
